# Safety of fluralaner oral solution, a novel systemic poultry red mite treatment, for chicken breeders’ reproductive performances

**DOI:** 10.1186/s13071-017-2480-2

**Published:** 2017-10-31

**Authors:** Bruno Huyghe, Gaelle Le Traon, Annie Flochlay-Sigognault

**Affiliations:** 1 MSD Animal Health Innovation SAS, 7 Rue O. de Serres CS 67131, 49071 Beaucouzé Cedex, France; 2 Merck Animal Health, 2 Giralda Farms, Madison, NJ 07940 USA

**Keywords:** Fluralaner, Breeder hens, Chickens, Safety, Poultry red mites, Drinking water, Parasiticide, Isoxazoline, Reproduction

## Abstract

**Background:**

Poultry mites are the most significant pest affecting production systems in the chicken egg-laying industry, altering the health condition of the birds, and causing stress, mortality and decline of egg quality impacting economic performance. Fluralaner is a novel systemic parasiticide that is effective against poultry mites (*Dermanyssus gallinae*, *Ornithonyssus sylviarum*) in chickens after oral administration. The evaluation of the safety of this new product in breeder chickens is particularly relevant because poultry mite infestation affects long cycle production systems, such as layers and breeders farms, for which the productivity heavily depends on the health of the reproductive function. This study was designed to investigate the safety for reproductive performances of fluralaner in male and female chickens at 3 times the recommended dose (1.5 instead of 0.5 mg/kg body weight) and 2 times the recommended duration (4 administrations instead of 2 administrations, with a 7 day interval between administrations).

**Methods:**

This randomized, parallel-group, blinded study included 432 Bovans brown parent stock chickens (48 males and 384 females, 17-week old). Birds were randomly assigned to 16 pens (three males and 24 females per pen), and then each pen assigned to one of the two treatment groups (8 pens, i.e. 216 birds per group). Fluralaner was administered via drinking water on a total of four occasions 7 days apart, at daily doses of 1.5 mg fluralaner/kg body weight, equivalent to 3 times the recommended dose of fluralaner per administration and 2 times the recommended number of administrations. Birds supplied with non-medicated drinking water served as controls. The treatments were given at time of peak egg production in the bird’s life: i.e. at 30 to 34 week of age. During that period, all adult chickens were clinically observed. The reproductive performances were carefully monitored including the number of eggs laid, egg weight, fertility and hatchability. Furthermore, the health and viability (up to 14 days of life) of randomly selected chicks was also monitored.

**Results:**

There were no clinical findings related to fluralaner treatment. There were no statistically significant differences between the reproductive performances of treated and control groups, nor in their progeny chickens viability.

**Conclusions:**

Oral administration of fluralaner was well tolerated by breeder chickens with a safety margin of approximately 3-fold obtained. Fluralaner had no effect on the egg number, weight and fertility, and no effect on egg hatchability or chick viability. Based on these results, a safe use of the new mite treatment proposed with fluralaner administered via drinking water is expected in layer and breeder field industrial conditions.

## Background

The poultry red mite (*Dermanyssus gallinae*) is a blood-sucking ectoparasite of domestic and feral birds, and is particularly important in commercial layers and breeders. Infestation by this parasite is associated with a decreased health condition [1], stress and decline of egg quality. Increased hen mortality is observed in severe infestations [2]. The egg production period (above 1 year) in the layers and breeders industry contributes to the establishment of strong parasite populations in facilities, which are difficult to eradicate. The replacement of traditional cages by enriched cages provides better hiding opportunities for poultry red mite (PRM) [3]. Consequently, PRM is widespread in commercial layer and breeder facilities [3]. Few products are licensed for use against mites in chickens [4]. Fluralaner is a novel systemic parasiticide, inhibitor of ligand-gated chloride channels (γ-aminobutyric acid (GABA) and L-glutamate gated chloride channels) with significant selectivity for arthropod neurons over mammalian neurons [5]. Fluralaner provides efficacy against infestation with PRM in chickens after oral administration via drinking water [6]. The recommended treatment dosage of fluralaner oral solution for chicken is two administrations of 0.5 mg/kg body weight, given at 7 days interval. The general safety of such fluralaner treatment has been investigated in laying hen by Prohaczik et al. [7]. The authors have shown that fluralaner is well tolerated in poultry, with a high margin of safety. However, fluralaner treatment against PRM is administered while birds are in lay of infertile eggs (commercial layers) or of fertile eggs (breeders), and no data are available on fluralaner impact on reproductive performances in any avian species.

This study was designed to investigate more specifically the safety for reproductive performances of this novel parasiticide, fluralaner, in male and female chickens at 3 times the recommended dose (1.5 instead of 0.5 mg/kg body weight) and 2 times the recommended duration (4 administrations instead of 2 administrations, with a 7 day interval between administrations). In this study, the treatments were given at time of peak egg production in the bird’s life, when the reproduction system is the most stressed.

## Methods

### General

This randomized, parallel-group, blinded study included parent stock chickens. Half of the chickens received fluralaner medicated water (treated group), the other half received standard water as negative control (control group). The study design was based on VICH GL 43 target animal safety requirements for veterinary pharmaceutical products [8]. This study was conducted in USA in compliance U.S. FDA Good Laboratory Practices (GLP) for Nonclinical Laboratory Studies 21 CFR Part 58.

### Subjects

Seventeen week-old Bovans brown parent stock chickens were used. A total of 648 individually identified birds (576 females and 72 males) were assigned to a total of 24 pens. Three males and 24 females were assigned per pen, reaching a male to female ratio of 11%, consistent with the 9–11% recommended for the breeder industry [9]. At inclusion, males weighed 1.9–2.05 kg and females weighed 1.35–1.45 kg. Neither vaccinations nor systemic medications were administered during the study.

### Breeding conditions for parents

Pens were 8.5 m^2^ (equivalent 0.29 m^2^/bird), and contained 4 egg nests, feeders (one for males and one for females) and drinking nipples. Wood shaving litter covered the pen floors. Housing conditions were in line with the Bovans management guide [10]. Pens and litter were inspected daily. Daily temperatures were recorded and ranged from 1.7 °C to 22.8 °C. Daily relative humidity was recorded and ranged between 12 and 85%. The minimum amount of light the animals were exposed to was measured weekly and ranged between 44.5–56.6 lx.

Birds were fed with feed according to their age, manufactured specifically for the study according to the Bovans management guide [10]. The same ration was proposed to males and females during the course of the study. The amount of feed proposed was controlled (as per management guide), and feed consumption recorded. Birds had free access to well water from 4 individual drinking nipples in each pen. For the water consumption measurement and treatment periods, the pipes connecting each pen nipple were derived for connection to a plastic can placed above each pen.

### Handling conditions for eggs and chicks

Part of the eggs laid during the study was allowed to hatch, and some of the derived chicks were monitored over 2 weeks post-hatching. Eggs allowed to hatch were incubated for 21 days: eggs were set for 18 days in a commercial incubator controlling temperature (36.7–38.9 °C) and humidity (62–69%), then the eggs were transferred and set for 3 days in a commercial hatcher controlling temperature (36.1–37.2 °C) and humidity (62–68%) to hatch the progeny. On the day of hatching, selected trays of eggs were briefly removed from the hatcher, evaluated, and then returned to the hatcher in order to monitor the progress of hatching at approximately two-hour intervals. Completion of hatching was determined based on the number/% of chicks that hatched, if the navel/yolk sac area was dry, and if the feathers were dry. Hatching was considered complete when approximately 95% of the hatched chicks had dry feathers, and all of the eggs were removed from the hatcher at the same time. When monitoring of hatched chicks was required, chicks were housed in a barn, daily temperatures and humidity were recorded and ranged between 18.9–35.6 °C and 18–81%, respectively.

### Study design

**Fig. 1:**
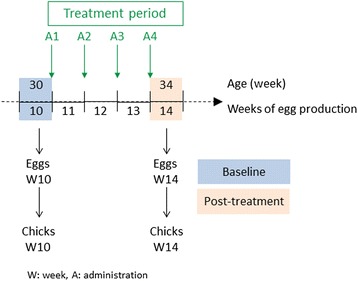
Study timelines

The different phases of the study are presented in Fig. 1.

#### Selection of treatment groups

From 17 to 29 weeks of age, the total 24 pens were monitored for egg production and general health. All the eggs laid during the 24th week of age were collected, and allowed to hatch. On week 30 of age, 16 pens were selected treatment, based on egg production, egg weights, daily observation records, and the highest number of live chicks hatched from eggs laid during the 24th week of age. After randomization of the 16 pens to the two treatment groups, all the eggs laid during the 30th week of age were collected, allowed to hatch and 32 of the hatched chicks per group were observed (body weight and clinical conditions) for 2 weeks post-hatching. These chicks data, together with adult data recorded during the 30th week of age (see Records section), were considered as baseline data.

#### Treatments

The 16 selected pens received either non-medicated well water (negative control group) or, fluralaner, formulated as a 10 mg/ml solution, diluted into drinking water at 3 times the recommended treatment dose, i.e. 1.5 mg fluralaner/kg body weight (treated group). Both groups received a total of 4 administrations (A1 to A4) 7 days apart, starting on first day of Week 11, until first day of Week 14. Four administrations correspond to two times the recommended duration of treatment. The treatment period (4 weeks) extended for at least one estrous cycle in females (1 day, [11]), one egg formation period (11 days, [11]), and one spermatogenic cycle in the males (13 days, [12]). On first administration day, the average body weight was 2.56 kg and 2.47 kg for the males, and 1.81 kg and 1.81 kg for the females, in the control and treated group respectively. The body weights of the parents were in line with the Bovans parent performance guide [13], for birds of that age.

For each treatment group, the water consumption of each pen was measured during 6 consecutive hours for 2 days prior to each administration. On each administration occasion, the medicated water to be provided to treated group was prepared by dilution of fluralaner 10 mg/ml solution into well water, based on (i) the target dose rate (1.5 mg fluralaner/kg body weight); (ii) its total body weight; and (iii) its overall pen 6-h water consumption. Confirmatory analyses of the batches of medicated water prepared were conducted to determine the actual concentrations of fluralaner supplied to pens. On each administration day, the amount of medicated water provided to each pen was weighed, as well as the amount remaining after 6 h, to determine the amount of medicated water ingested actually by each pen. The actual fluralaner doses consumed per pen were calculated. The pens from the control group received non-medicated well water. Analysis confirmed that no fluralaner was detected in any of the batches of water provided to the chickens from the control group. At each administration date, the mean fluralaner dose per pen (treated group) achieved was 1.4 mg/kg (corresponding to 2.8 times the recommended dose).

#### Records

General health observation of the birds (including external examination of the feathers, eyes, and beaks, as well as any abnormalities in behavior, locomotion and mortality) were performed twice daily during the course of the study, for adults and chicks.

Between week 10 to 15 of production the birds were weighed weekly by pen.

The following variables were recorded/ calculated per pen:

(i.) number of eggs laid (daily);
(ii.) individual egg weight (daily);
(iii.) fertility at incubation day 7 (viable eggs);
(iv.) hatchability (on incubation day 21): total (number of chicks / number of eggs transferred ×100), and viable (number of chicks / number of viable eggs × 100);
(v.) chick viability on newly hatched chicks (on incubation day 21), based on the following binomial variables: stance, deformity, navel aspects, hydration, down feather: color, length (continuous variables) and dryness;
(vi.) chick viability at 14 days old (post hatch): mortality rate and based on the following binomial variables: stance, deformity, navel aspects, hydration, down feather: color, length (continuous variables) and dryness;
(vii.) chick body weight (at hatch and at 14 days old).

### Analysis of the results

The study data were evaluated for fluralaner-related effects on the reproductive safety parameters by comparison to the negative control group. The parameters of primary importance in this study were: fertility, hatchability, and chick viability/survival. The study design did not provide for differentiating effects specific to the male or the female. The reproductive parameters collected before treatment period (Week 10, baseline) and at the last week of the treatment period (Week 14, post-treatment) were statistically compared between groups. The duration of the treatment period (4 weeks) ensured that eggs collected for hatching during the last week of the treatment period were issued from ovum and sperm exposed to fluralaner during their formation.

All statistical comparisons were performed at the 0.1 level of significance. Statistical analyses were performed using SAS version 9.4. The pen was considered as the statistical unit and used as random effect in the statistical analysis. Treatment, time and sex were used as fixed effects. Data from Week 10 served as baseline.

The following variables were analyzed with a repeated measures analysis of covariance: feed consumption, water consumption, pen weight, mean egg number and mean egg weight. Chick hatch weight, chick feather length, and 14-day old chick weight were analyzed with an analysis of variance. Finally, linear model was used for adult mortality, egg fertility binomial variables (presence of deformity, navel healed …) and 14-day old chick survival analysis.

## Results and discussion

The hen day egg production was 94.2–96.9% (1266 to 1303 eggs were laid per group during Week 10 or Week 14 for 1344 possible eggs at a 100% hen day egg production). These figures are higher than the production standard proposed by Meijerhof [9] for breeders. The hatching per egg ranged between 80.1–84.8%; in line with the 78–90% interval for 30–34 weeks-old breeders proposed in Meijerhof [9]. These production figures attest to the representativeness of the study for the breeder industry. The number of eggs and chicks per group are presented in Table 1.

**Table 1:** Number of eggs and chicks per group

	Egg laying period^a^	Negative control group		Fluralaner-treated group	
		*n*	% per hen day	*n*	% per hen day
Eggs laid	Baseline	1266	94.2	1280	95.2
	Post-treatment	1294	96.3	1303	96.9
Chicks hatched	Baseline	1085	80.7	1076	80.1
	Post-treatment	1130	84.1	1140	84.8

^a^Baseline is Week 10 (pre-treatment); post-treatment is Week 14, after the 4th treatment

Water consumption was not affected by fluralaner administrations (ANCOVA: *F*_(1,13.2)_ = 0.00, *P* = 0.9622). This is consistent with the results of Prohaczik et al. [7] where no effect on water consumption was detected up to 5× the recommended dose. Fluralaner administrations had no detectable effect on feed consumption. No statistical analysis was performed on feed consumption as 100% of the feed was consumed in all pens, regardless of the group. There was no mortality observed during the study among the adult birds, and no treatment-related effects on adult bird clinical observations or pen weights.

**Fig. 2:**
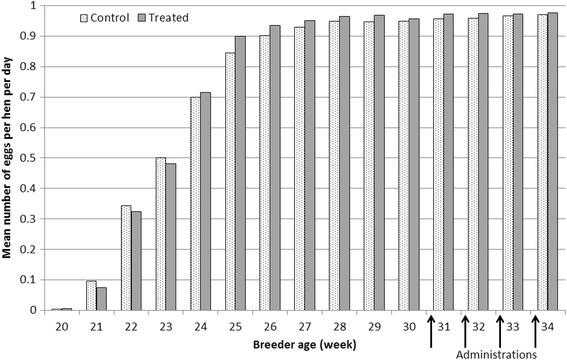
Weekly mean number of eggs per hen per day per group

**Fig. 3:**
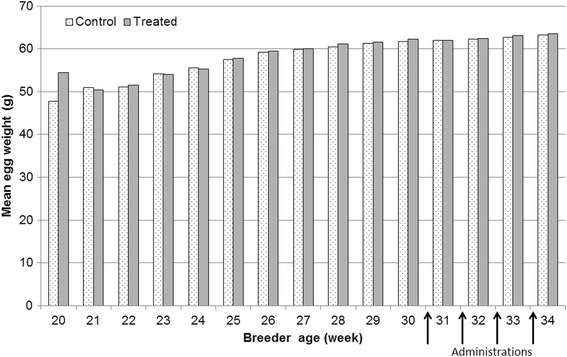
Weekly mean egg weight per group

No statistically significant differences were detected between groups for egg production and for egg weights. Egg production and egg weights are presented in Figs. 2 and 3.

There were no treatment-related differences in egg fertility and in egg hatchability. Fertility was between 96.7–97.4% in both groups at Week 10 and at Week 14. Hatchability was between 84.1–87.5% in both groups at Week 10 and at Week 14. Egg fertility and egg hatchability results are presented in Table 2.

**Table 2:** Fertility, hatchability and viability per group

Variable	Egg laying period^a^	Negative control group		Fluralaner-treated group		*P*-value
		Mean	SD	Mean	SD	
Fertility and hatchability						
Fertile eggs/ Eggs laid	Baseline	0.973	0.004	0.974	0.004	0.9291
	Post-treatment	0.967	0.006	0.968	0.006	
Chicks hatched/ Eggs laid	Baseline	0.857	0.01	0.841	0.009	0.9193
	Post-treatment	0.873	0.004	0.875	0.008	
Chick viability variables						
Down feather length (cm)	Baseline	1.118	0.007	1.084	0.007	0.5320
	Post-treatment	1.043	0.006	1.038	0.005	
Newly hatch chick weight (g)	Baseline	40.89	0.106	40.52	0.111	0.9463
	Post-treatment	41.7	0.106	41.41	0.114	
Female Day 14 chick weight (g)	Baseline	119.3	2.581	112.4	3.911	0.5682
	Post-treatment	106.1	4.273	104.1	2.862	
Male Day 14 chick weight (g)	Baseline	128.5	2.127	122.9	3.166	
	Post-treatment	105.9	4.741	112.1	2.077	

^a^Baseline is Week 10 (pre-treatment); post-treatment is Week 14, after the 4th treatment

*Abbreviation*: *SD* standard deviation

There were no treatment-related differences in chick viability (newly hatched or at 14 days old) and chick clinical observations. Chicks’ mortality at 14 days (from eggs laid week 14) was 0% in both the treated and control groups. For chick viability binomial variables (newly hatched or at 14 days-old), the majority of responses were the same and abnormal data were too sparse for analysis (only one chick (control group) from Week 10 (baseline) was abnormal for all binomial viability variables). Newly hatched chicks weight at week 14 chicks was 41.7 ± 0.1 g and 41.4 ± 0.1 g in the control and the fluralaner-treated groups respectively. Fourteen days later, the chicks had grown to 106.1 ± 4.3 g and 104.1 ± 2.9 g for females and 105.9 ± 4.7 g and 112.1 ± 2.1 g for males, in the control and the fluralaner-treated groups respectively. No differences were detected between groups for chick weight (at hatching or at 14 day-old) and down feather length. Results are presented in Table 2.

## Conclusion

This detailed evaluation of the reproduction safety of fluralaner, a novel systemic parasiticide drug, following oral administration at doses much higher than the recommended treatment dose, did not reveal any adverse effects in laying breeder hens. Oral administration of fluralaner via drinking water to laying hens at dose rates of up to 3× the recommended dose and 2 times the recommended treatment duration did not lead to any treatment-related findings that could be detected through careful clinical observations of reproductive performance of parents and health of the progeny chickens. Oral administration of fluralaner via drinking water was well tolerated by breeder chickens, a safety margin of approximately 3 was demonstrated. Based on the present results, the use of the new fluralaner treatment against mites, administered via drinking water, is expected to be safe for breeder chickens under field industrial conditions, and to have no negative impact on their reproduction.

## Abbreviations

CFR: Code of Federal Regulation
FDA: Food and Drug Administration
GABA: γ-aminobutyric acid
GLP: Good laboratory practices
PRM: Poultry red mite
VICH: Veterinary International Committee on Harmonization

## References

[1] Chauve C. The poultry red mite *Dermanyssus gallinae* (de Geer, 1778): current situation and future prospects for control. Vet Parasitol. 1998;79:239–45. 10.1016/s0304-4017(98)00167-8 9823064

[2] Cosoroaba I (2001). Massive *Dermanyssus gallinae* invasion in battery-husbandry raised fowls. Rev Med Vet.

[3] Sparagano O, Pavlicevic A, Murano T, Camarda A, Sahibi H, Kilpinen O (2009). Prevalence and key figures for the poultry red mite *Dermanyssus gallinae* infections in poultry farm systems. Exp Appl Acarol.

[4] Sparagano OA, George DR, Harrington DW, Giangaspero A (2014). Significance and control of the poultry red mite *Dermanyssus gallinae*. Annu Rev Entomol.

[5] Gassel M, Wolf C, Noack S, Williams H, Ilg T (2014). The novel isoxazoline ectoparasiticide fluralaner: selective inhibition of arthropod γ-aminobutyic acid- and L-glutamate-gated chloride channels and insecticidal/acaricidal activity. Insect Biochem Mol Biol.

[6] Heckeroth AR, Zoller H, Flochlay-Sigognault A, Huyghe B (2015). Use of isoxazoline derivatives for the treatment or prevention of arthropod infestations in poultry. Patent WO.

[7] Prohaczik A, Menge M, Huyghe B, Flochlay-Sigognault A, Le Traon G (2017). Safety of fluralaner oral solution, a novel systemic antiparasitic treatment for chickens, in laying hens after oral administration *via* drinking water. Parasit Vectors.

[8] VICH GL 43: Target Animal Safety for Veterinary Pharmaceutical Products. 2008, Belgium, URL: http://www.vichsec.org/pdf/0708/GL43-st7.doc. Accessed 1 Aug 2017.

[9] Meijerhof R, Bell DD, Weaver WD (2002). Managing the breeding flock. Commercial chicken meat and egg production 5th edition volume II.

[10] ISA Poultry, Bovans Brown General Management Guide Parent Stock. 2009, URL: https://kenanaonline.com/files/0082/82008/General%20Management%20Guide%20Parent%20Stock%20ISA%20brown.pdf. Accessed 1 Aug 2017.

[11] Bell D, Bell DD, Weaver WD (2002). Formation of the egg. Commercial chicken meat and egg production 5th edition volume II.

[12] Etches R. J. The male. In: Etches R. J., editor. Reproduction in poultry. CAB International, 1996. p. 208–233.

[13] ISA Poultry, Bovans Brown Parent Stock Product Performance. 2007, URL: http://webpticeprom.ru/download/spravochniki/Bovans_Browh/5.pdf. Accessed 1 Aug 2017.

